# Association between fractures and traditional risk factors for osteoporosis and low bone mineral density in patients with obesity

**DOI:** 10.20945/2359-3997000000331

**Published:** 2021-02-25

**Authors:** Letícia Guadanhim Sampaio, Janaina Marques, Ricardo Rasmussen Petterle, Carolina Aguiar Moreira, Victoria Zeghbi Cochenski Borba

**Affiliations:** 1 Universidade Federal do Paraná Curitiba PR Brasil Universidade Federal do Paraná (UFPR), Curitiba, PR, Brasil.; 2 Universidade Federal do Paraná Curitiba PR Brasil Setor de Ciências da Saúde, Universidade Federal do Paraná (UFPR), Curitiba, PR, Brasil.; 3 Universidade Federal do Paraná Departamento de Medicina Interna Curitiba PR Brasil Departamento de Medicina Interna, Universidade Federal do Paraná (UFPR); Serviço de Endocrinologia e Metabologia do Hospital de Clínicas da UFPR (SEMPR), Curitiba, PR, Brasil.

**Keywords:** Bone mineral density, osteoporosis, fracture, obesity, proton pump inhibitors

## Abstract

**Objective:**

To evaluate the reasons for request of bone mineral density (BMD) evaluation and correlate the BMD results with previous fractures, risk factors for osteoporosis, and clinical characteristics in patients with obesity.

**Subjects and methods:**

Cross-sectional, retrospective, single-site study including adult patients with body mass index (BMI) ≥ 30 kg/m^2^ and BMD evaluation between January 2015 and May 2016 selected from a BMD database. Data on demographic characteristics, lifestyle habits, comorbidities, medications, risk factors, previous fractures, and indications for BMD evaluation were collected from the participants’ medical records.

**Results:**

The study included 619 patients (89.9% women, mean BMI 34.79 ± 4.05 kg/m^2^). In all, 382 (61.7%), 166 (26.8%), and 71 (11.5%) patients had class 1, 2, and 3 obesity, respectively. The most frequent (29.9%) reason for BMD evaluation was for osteoporosis monitoring. In all, 69.4% of the patients had low BMD. Multivariate analysis showed that age, calcium supplementation, and previous osteoporosis or osteopenia were associated with low BMD, while age, vitamin D supplementation, use of proton pump inhibitors (PPIs), and low BMD were associated with previous fractures (p < 0.05 for all).

**Conclusions:**

Among patients with obesity identified from a tertiary hospital database, those with low bone mass and risk factors traditionally associated with fractures had an increased history of fractures. Patients with greater BMI had better bone mass and fewer fractures. These findings indicate that the association between reduced weight, risk factors for osteoporosis, and fractures remained despite the presence of obesity in our population.

## INTRODUCTION

Obesity and osteoporosis are major health concerns that are interrelated on many levels. Increased body mass index (BMI) has a positive effect on bone mineral density (BMD) and protects against the development of osteoporosis and occurrence of fractures (1,2). The greater mechanical loading and strain from the increased fat and lean mass in individuals with higher BMI increases bone strength and reduces the risk of fractures. In contrast, overweight and visceral fat may also increase the risk of fractures due to inflammation and bone resorption (3–5). Although these associations have been pointed out in different studies, they have not been largely explored in the Brazilian population.

In 2014, the World Health Organization (WHO) estimated that obesity affected 24% and 17% of the Brazilian women and men, respectively (6). These rates are alarming, considering that the corresponding global rates of obesity were 15% and 11% in women and men in that same year. A survey sponsored by the Brazilian government found a 7% increase in obesity rates over 10 years, which was more prominent among women. The survey also pointed out that the prevalence of obesity doubles after 25 years and increases with aging until the age of 64 years, after which it declines slightly (7). Obesity is commonly associated with physical inactivity and several comorbidities, which may contribute to an increased risk of fractures (8).

The aging of the population has increased the prevalence of osteoporosis in older individuals (5,9), and due to these individuals’ greater susceptibility to falls and fractures, has heightened the burden on the public health care system. Since fractures are a major cause of morbidity and mortality in postmenopausal women, prevention and diagnostic screening of this condition using bone densitometry is strongly recommended in this population (10,11). Still, the risk factors for fractures among obese individuals with available BMD data in the Brazilian population is largely unknown.

Based on the considerations above, the aim of this study was to evaluate the indications for BMD evaluation in patients with obesity following up at a tertiary hospital, and evaluate the correlation between the degree of obesity with BMD results, previous fractures, risk factors for osteoporosis, and comorbidities.

## SUBJECTS AND METHODS

The protocol for this retrospective, cross-sectional, single-site study was approved by the Human Research Ethics Committee of the *Hospital de Clínicas* at *Universidade Federal do Paraná* (HC-UFPR; CAAE: 51231215.5.0000.0096).

We selected the study sample from a database of patients who had undergone bone densitometry between January 2015 and May 2016 at the Endocrinology and Metabolism Unit (*Serviço de Endocrinologia e Metabologia*, SEMPR) of HC-UFPR. We included all patients aged ≥ 18 years and with BMI ≥ 30 kg/m², and excluded individuals weighing > 120 kg, participating in ongoing clinical trials, or with discrepant weight and height values in the medical records and in measurements obtained before dual-energy X-ray absorptiometry (DXA) scanning.

We collected information from the patients’ medical records including demographic data (date of birth, sex, and race), weight and height measurements, lifestyle habits (smoking and alcoholism), indications for BMD evaluation, presence of comorbidities, use of medications, history and type of previous fractures (low or high impact), and results of laboratory tests. Considering that this was a retrospective analysis, we were unable to collect data on the participants’ calcium intake or body composition.

We considered for the analysis the results of laboratory tests collected closest to the date of the BMD evaluation. The tests included glycated hemoglobin (colorimetric method, normal value [NV] < 6%), high-density lipoprotein (HDL) cholesterol (colorimetric method, NV = 40–80 mg/dL), total cholesterol (colorimetric method, NV ≤ 200 mg/dL), triglycerides (colorimetric method, NV = 50–150 mg/dL), low-density lipoprotein (LDL) cholesterol calculated with the Friedewald equation (NV = 85–125 mg/dL), parathyroid hormone (PTH; chemiluminescence, NV = 15–68.3 pg/mL), free thyroxine (NV = 0.7–1.48 ng/dL), thyroid-stimulating hormone (TSH; NV = 0.35–4.94 pg/mL), vitamin D (25OHD; LIAISON 25 OH Vitamin D Total Assay, DiaSorin S.p.A., Saluggia, Italy); alanine aminotransferase (ALT; enzimatic reaction, NV = 0–55 U/L), aspartate aminotransferase (AST; enzimatic reaction, NV = 5–34 U/L), calcium (Arzenazo III, NV = 8.5–10.2 mg/dL), creatinine (Jaffé method, NV = 0.8–1.3 mg/dL), and fasting glucose (hexokinase/glucose-6-phosphate-dehydrogenase method, NV < 100 mg/dL).

Based on 25OHD levels, the vitamin D levels were categorized as deficient (< 20 ng/mL), insufficient (20–29 ng/mL), and normal (≥ 30 ng/mL) (12).

Patients with elevated PTH and calcium levels or with PTH levels inappropriately high for serum calcium levels and vitamin D sufficiency were classified as having primary hyperparathyroidism, while those with elevated PTH in the presence of normal or low levels of calcium and vitamin D or individuals with chronic kidney disease were classified as having secondary hyperparathyroidism (13).

Bone mineral density was evaluated by DXA (Lunar Prodigy Advance, GE Medical Systems Lunar, Madison, WI, USA; weight limit 120 kg). Lumbar spine (L1–L4), total hip, and femoral neck BMD was analyzed according to the criteria defined by the WHO and International Society for Clinical Densitometry (ISCD) and categorized as normal, low (according to age), osteopenia, and osteoporosis. Information on the participants’ weight and height were obtained before DXA scanning, and the results were compared with the corresponding values from medical records. Weight was measured on a Welmy W200/5 scale (Welmy, São Paulo, Brazil) with the participants wearing light clothes, and height was measured on a wall-mounted stadiometer (Ayrton Stadiometer Model S100, Ayrton Corporation, Prior Lake, MN, USA).

We measured BMI as weight (kg) divided by squared height (m^2^), and categorized the degree of obesity of the patients based on BMI results as class 1 (30.0–34.9 kg/m^2^), 2 (35.0–39.9 kg/m^2^), or 3 (BMI > 40 kg/m^2^) (7).

### Statistical analysis

All data were analyzed using the software R, version 3.4.4 (The R Foundation for Statistical Computing, Vienna, Austria). Fisher's exact test and chi-square test evaluated the association between two qualitative variables. Comparisons between groups were performed using Student's *t* test, nonparametric Mann-Whitney test, or nonparametric Kruskal-Wallis test, depending on the distribution and number of groups. The Tukey test was performed for multiple comparisons. The normal distribution of continuous variables was assessed with the Shapiro-Wilk test.

For multivariate analysis, a logistic regression model was adjusted considering the presence or absence of fractures or low BMD as dependent variables and using as explanatory variables those variables with p values < 0.05 in the univariate analysis. The results of this analysis are expressed as odds ratios (ORs) and 95% confidence intervals (CIs). A negative binomial regression model was fitted to identify the variables associated with a greater number of fractures.

Quantitative variables are summarized as mean (± standard deviation) values or median (minimum–maximum) values. P values < 0.05 indicated statistical significance.

## RESULTS

Of 2188 patients who underwent DXA scanning during the study period, 627 (28.6%) had obesity and met the criteria for inclusion in the study. Of these, 8 were excluded due to discrepant weight and height data obtained from medical records versus before DXA scanning. The final sample of 619 patients had a median age of 63 years (19–88 years) and comprised mostly of women and white individuals.

The patients were distributed according to the degree of obesity into classes 1 (n = 382, 61.7%), 2 (n = 166, 26.8%), and 3 (n = 71, 11.5%). Table 1 presents the demographic data and clinical characteristics of the study participants.

**Table 1:** Clinical and demographic data of the study participants

		N = 619 (%)
Age, years – median (range)		63.0 (19 – 88)
Sex		
	Men	62 (10.0%)
	Women	557 (89.9%)
Race		
	White	586 (94.6%)
	Black	32 (5.2%)
	Asian	1 (0.2%)
Weight, kg – mean ± SD		83.9 ± 13.0
Height, meters – mean ± SD		1.55 ± 0.08
BMI, kg/m² – mean ± SD		34.79 ± 4.06
Menopause		486 (87.6%)*
Current smoking		38 (6.1%)
Current alcohol intake		11 (1.7%)
BMD		
	Low bone mass	430 (69.4%)
	Bone mass below the expected value for age	14 (2.3%)
	Osteopenia	287 (46.4%)
	Osteoporosis	129 (20.8%)

BMI: body mass index; SD: standard deviation; BMD: bone mineral density

* Percentage of the total number of women (n=557).

The most prevalent comorbidities in our study sample were hypertension (n = 490, 79.1%) and dyslipidemia (n = 437, 70.5%), followed by type 2 diabetes mellitus (n = 331, 53.4%), osteopenia or osteoporosis (n = 317, 51.2%), and thyroid disease (n = 308, 49.7%). The medications more frequently used by the patients were vitamin D (n = 455, 73.5%) and antihypertensive agents (n = 447, 72.2%), followed by statins (n = 399, 64.4%), diuretics (n = 332, 53.6%), and oral antidiabetic agents (n = 332, 3.6%) (Supplementary Table 1).

### Laboratory tests

The median 25OHD level was 29.5 ng/mL (2–73.4 ng/mL). In all, 65 (16.7%) patients had vitamin D deficiency (median 17.4 ng/mL, range 2–19.9) and 138 (35.4%) had vitamin D insufficiency (median 25.7 ng/mL, range 20–29.9), while 186 (47.8%) individuals had normal vitamin D levels (median 37.4 ng/mL, range 30–73.4). Median levels of calcium and PTH were 9.5 mg/dL (1.14–14 mg/dL) and 70.1 pg/dL (2–968.3 pg/dL), respectively. Overall, 60 (9.6%) patients had hyperparathyroidism, including 21 (35%) with primary hyperparathyroidism and 37 (61.66%) with secondary hyperparathyroidism (8 with chronic kidney disease and 29 with vitamin D insufficiency). The type of hyperparathyroidism could not be determined in 2 patients.

The results of laboratory tests were comparable across different degrees of obesity, except in the group with obesity grade 1, which had greater mean total cholesterol (p=0.030) and LDL cholesterol (p=0.040) levels (Supplementary Table 2).

### Degree of obesity

The groups divided according to the degree of obesity differed in terms of age, race, low BMD, use of medications (antidiabetic drugs, aspirin, antidepressants, diuretics, and antiresorptive agents), comorbidities (menopause, diabetes, asthma, osteoporosis, previous bariatric surgery, psychiatric, and thyroid disease), and levels of total cholesterol and LDL cholesterol (p < 0.05 for all), as shown in Supplementary Table 2. Indeed, older patients (p = 0.003), white race (p = 0.031), taking calcium supplements (p = 0.040), in use of antiresorptive agents (p < 0.001), with prior fractures (p = 0.017), osteoporosis (p < 0.001), and neoplasia (p = 0.047) had lower BMI values (Supplementary Table 2). In contrast, individuals using antidiabetic drugs (p < 0.001), aspirin (p < 0.001), antidepressants (p = 0.002), or diuretics (p < 0.001) and those with asthma (p = 0.007), diabetes (p < 0.001), psychiatric illness (p = 0.043), and previous bariatric surgery (p = 0.020) had higher BMI values.

### Bone mineral density

The reasons for bone densitometry evaluation were identified in the medical records of 518 (83.7%) patients. The main reasons for requesting bone densitometry included monitoring of previously diagnosed osteopenia or osteoporosis (29.9%), presence of an endocrine or genetic condition (17.8%), and advanced age (9.0%). The BMD results were normal in 189 (30.5%) patients and low in 430 (69.4%) of them; of the latter, 287 (46.4%) were classified as osteopenia and 129 (20.8%) as osteoporosis. A total of 14 (2.3%) patients younger than 50 years had a BMD result lower than the value expected for their age group.

**Figure 1:**
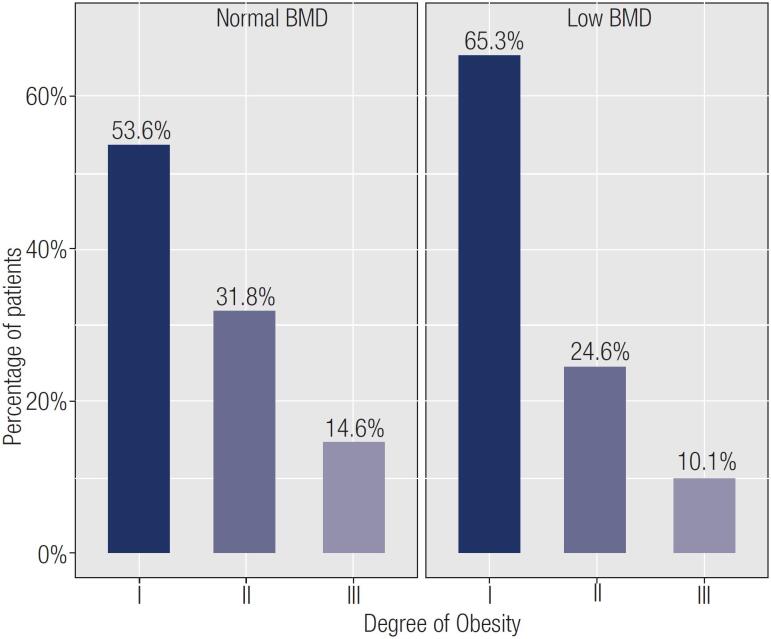
Bone mineral density according to the obesity class. BMD: bone mineral density.

In the overall sample, low BMD was more prevalent in older individuals (p < 0.001), with lower BMI (p = 0.001), previous fracture (p < 0.001), greater number of fractures (p < 0.001), use of vitamin D (p < 0.001), calcium intake (p < 0.001), use of antiresorptive drugs (p < 0.001), previous diagnosis of osteopenia (p = 0.014) or osteoporosis (p < 0.001), hyperparathyroidism (p = 0.002), dyslipidemia (p = 0.005), and higher PTH levels (p = 0.002). Low BMD was also more prevalent in patients with obesity class 1 followed by classes 2 and 3 (p = 0.019) (Figure 1). On multivariate analysis considering low BMD as a dependent variable and presence or absence of all significant explanatory variables as independent variables, age (OR 1.06, 95% CI 1.04–1.08), calcium intake (OR 2.55, 95% CI 1.59–4.14), and previous diagnosis of osteopenia (OR 15.66, 95% CI 7.9–34.84) or osteoporosis (OR 19.77, 95% CI 9.29–49.07) influenced the BMD result. The same analysis stratified by sex showed that in men, only age (OR 1.05, 95% CI 1.01–1.09) influenced BMD, while in women, BMD was influenced by age (OR 1.04, 95% CI 1.01–1.07), calcium intake (OR 1.85, 95% CI 1.01–3.45), PTH level (OR 1.01, 95% CI 1.008–1.02), and previous diagnosis of osteopenia (OR 19.33, 95% CI 8.12–54.54) or osteoporosis (OR 28.22, 95% CI 11.56–85.28).

### History of fractures

A total of 87 (14%) patients had a history of previous fractures, with a median number of fractures per patient of 1 and a maximum of 6. The use of vitamin D, calcium, antiresorptive agents, analgesics, and proton pump inhibitors (PPIs), along with lower BMD levels (at any site), age, and lower BMI were associated with a higher prevalence of fractures (Table 2). Of note, the results remained unchanged after excluding patients with glucose intolerance or diabetes.

**Table 2:** Distribution of variables according to the presence of a fracture

Variable	With fracture n (%) (n = 87)	Without fracture n (%) (n = 532)	P value
Age	70 (62 – 78)	63 (56 – 70)	p < 0.001
BMI (kg/m^2^)	33.8 ± 3.4	35 ± 4.1	p = 0.017
Vitamin D supplements	78 (89.7%)	9 (1.7%)	p < 0.001
Calcium supplements	57 (65.5%)	30 (5.6%)	p < 0.001
Calcium plus vitamin D supplements	79 (90.8%)	8 (1.5%)	p < 0.001
Bisphosphonates	51 (58.6%)	36 (6.8%)	p < 0.001
Osteoporosis treatment	81 (93.1%)	6 (1.1%)	p < 0.001
PPI	42 (48.3%)	45 (8.5%)	p < 0.001
Diuretics	54 (62%)	33 (6.2%)	p = 0.088
Analgesics	19 (21.8%)	68 (12.8%)	p = 0.048
Antidepressants	17 (19.5%)	70 (13.2%)	p = 0.129
Aspirin	18 (20.7%)	69 (13%)	p = 0.058
Insulin	13 (14.9%)	74 (13.9%)	p = 0.075
Antidiabetics drugs	33 (37.9%)	54 (10.1%)	p = 0.028
BMD			p < 0.001
Normal	9 (1.45%)	180 (29.07%)	
Below the expected value for age	4 (6.94%)	10 (1.61%)	
Osteopenia	43 (6.94%)	244 (39.41%)	
Osteoporosis	31 (5.00%)	98 (15.83%)	

n: number; BMI: body mass index; PPI: proton pump inhibitors; FN: femoral neck; BMD: bone mineral density. Osteoporosis treatment comprised vitamin D, calcium, and bisphosphonates. Statistical significance was set at p < 0.05.

The prevalence of fractures across groups divided according to the degree of obesity was 71.3% (n = 62) in class 1, 23% (n = 2) in class 2, and 5.7% in class 3 (n = 5; comparison of all three groups p = 0.274). A history of previous fractures (p = 0.091) and the number of fractures (p = 0.101) were not associated with the degree of obesity.

**Figure 2:**
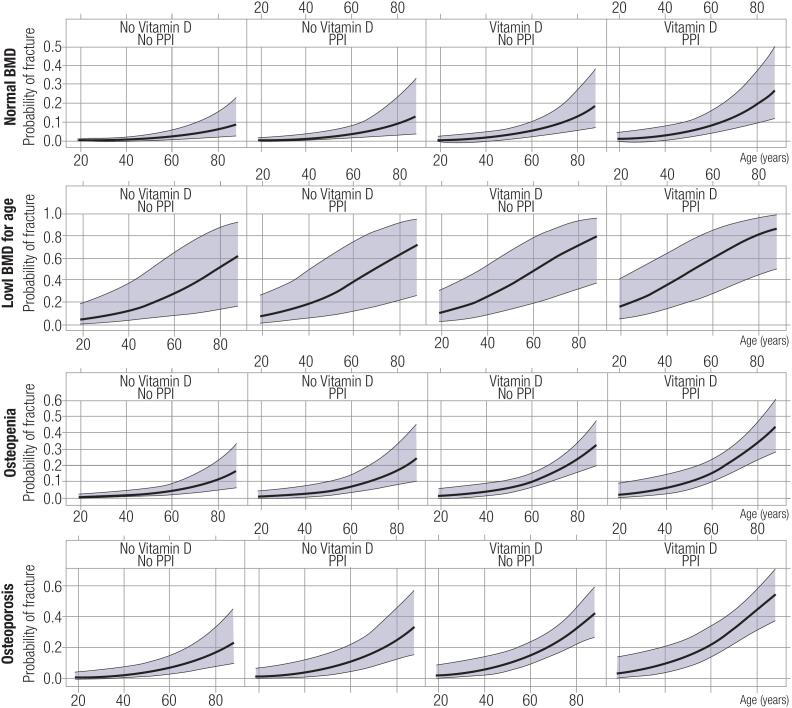
Binary logistic regression analysis showing the probability of fractures considering the presence or absence of all variables identified as significant in the univariate analysis. PPI: proton pump inhibitors.

On multivariate analysis considering the presence of fractures as a dependent variable and including as independent variables the presence or absence of the significant explanatory variables age, BMI, use of vitamin D, use of PPIs, and final BMD result, all variables, except for BMI, influenced the prevalence of fractures. The odds of a patient having a previous fracture was 16.6 times greater when his or her BMD result was below the expected value for age (versus normal for age), 3.2 times higher with (versus without) a previous diagnosis of osteoporosis, 1.62 times higher in patients taking (versus not taking) PPIs, and 2.38 times higher in those taking (versus not taking) vitamin D. An analysis with other variables fixed in the logistic regression model estimated a 5% increase in fracture risk with each year increase in patient's age (Table 3 and Figure 2). The presence of osteopenia and use of vitamin D and PPIs were no longer significant in a separate analysis excluding patients with diabetes.

**Table 3:** Variables associated with previous history of fractures

Variables	OR (95% CI)	P value
Age	1.05 (1.02 – 1.08)	< 0.001
Use of vitamin D supplements	2.38 (1.19 – 5.26)	0.022
Use of proton pump inhibitors	1.62 (1.01 – 2.36)	0.047
BMD below the expected value for age	16.58 (3.39 – 76.42)	< 0.001
Osteopenia	2.08 (0.99 – 4.80)	0.067
Osteoporosis	3.22 (1.45 – 7.76)	0.006

OR: odds ratio; CI: confidence interval; Model: binary logistic regression.

### Number of fractures

The number of fractures was associated with the use of vitamin D, calcium, and bisphosphonates alone or combined, use of PPIs or antidiabetics, presence of osteoporosis, low BMD, and history of bariatric surgery. The number of fractures correlated positively with age (p < 0.001, r = 0.186) and negatively with weight (p < 0.001, r = −0.158), BMI (p = 0.029, r = −0.087), and BMD at the lumbar spine (p < 0.001, r = −0.197), femoral neck (p < 0.001, r = −0.204), and total hip (p < 0.001, r = −0.228).

A negative binomial regression analysis of the factors associated with a greater number of fractures and including all variables identified as significant in the univariate analysis showed that age, diagnosis of osteoporosis, treatment of osteoporosis, and use of antidiabetic drugs were associated with an increased number of fractures, while previous bariatric surgery and greater spinal BMD decreased this risk (Table 4).

**Table 4:** Variables associated with a greater number of fractures

Variables	Relative Risk	CI (2.5 – 97.5%)
Age	1.031	1.008 – 1.055
Previous osteoporosis	1.976	1.142 – 3.415
Osteoporosis treatment	2.232	1.046 – 4.759
Use of antidiabetic drug	0.598	0.366 – 0.976
Previous bariatric surgery	0.220	0.062 – 0.786
Greater spinal BMD (g/cm^2^)	0.129	0.027 – 0.613

CI: confidence interval; BMD: bone mineral density; Model: negative binomial regression.

## DISCUSSION

In this study, we analyzed 619 patients with obesity and BMD data following up at a tertiary endocrinology center. Due to available clinical data of the patients, we were able to analyze the impact of BMI on BMD and prevalence of fractures.

Most patients in our study (89.9%) were women, allowing us to directly compare our data with those from other studies investigating the influence of BMI in populations comprising exclusively women (3,14). The study sample was also representative of the obese population in southern Brazil, which comprises mostly women (7).

The recommendations for DXA scanning in our study participants (*i.e.,* age, presence of risk factors, and previous metabolic bone disease) are aligned with the ISCD recommendations for obtaining DXA scanning (15).

The high rate of comorbidities and frequent use of medications in our study participants may be explained by the strong association between obesity and more than 20 comorbidities (8,16) and the fact that the study was conducted at a tertiary hospital. The substantial use of antidepressant agents by individuals with obesity, as observed among individuals with obesity classes 2 and 3 in the present study, has been previously described (17) and seems to be worse the greater the BMI (18).

Almost 70% of our study sample had low BMD and 20% had osteoporosis. Low BMD was associated with traditional risk factors for osteoporosis and fractures, which is aligned with findings from recent studies (19,20); however, only age, calcium intake, and previous diagnosis of osteopenia or osteoporosis remained significant in the multivariate analysis, even when patients with diabetes were excluded, suggesting that not all traditional risk factors apply to patients who are obese. On the other hand, normal BMD was associated with greater BMI, which is in agreement with the literature (20,21).

If, on the one hand, obesity is associated with greater BMD, it is also associated with an increased risk of falls and may not protect against all fractures, as initially believed (22). Also, the protective effect from the increased BMD may not be sufficient to overcome the risk of fracture from all factors involved in falls among individuals with obesity (23). These individuals present fewer fractures classically related to osteoporosis, but in turn, have an increased frequency of nonvertebral fragility fractures such as those in the proximal humerus, upper legs, and ankles. Although these types of fractures were not reported in our sample, the presence of a history of fracture was associated with traditional risk factors for fractures and increased number of fractures such as age, use of PPIs, low BMD, and history of osteoporosis.

Our results differ from those of previous studies that have shown a paradoxical effect of excessive fat mass (from obesity) increasing the risk of osteoporosis and fractures (4,5). Indeed, the occurrence of fractures in the present study was not associated with the degree of obesity. This discrepancy may be partially explained by limitations of BMI in distinguishing between lean and fat mass (24). A meta-analysis has shown that the relationship between BMI and fracture risk follows a U-shaped curve, confirming a nonlinear effect of BMI on fracture risk, with a higher prevalence of fractures at the two BMI extremes (25). However, several studies, including the present one, have already reported this paradox (4,21,26). The Global Longitudinal Study of Osteoporosis in Women (GLOW) found that among women with previous fractures, treatment for osteoporosis was more frequent among those with lower BMI compared with those with obesity (27). This was also observed in the present study, where class 1 obesity was associated with increased use of medications for treatment of osteoporosis.

Increased body weight has been associated with delayed bone loss at menopause. However, the Fracture Liaison Service has reported an unexpected increase in the prevalence of obesity (27%) in postmenopausal women with fragility fractures (14). Another complication of class 2 and 3 obesity is loss of cortical bone (28) associated with secondary hyperparathyroidism (29). Complications associated with obesity may also influence the quality of life and risk of fractures (4,20). Surprisingly, individuals not receiving vitamin D in the present study had a lower fracture rate compared with those receiving this vitamin. This may be explained by the fact that the study was conducted at a tertiary hospital, in which patients with osteoporosis were already receiving vitamin D as part of their treatment for this condition and that our sample included many patients with secondary hyperparathyroidism in whom the vitamin D deficiency was probably already being treated, which is similar to the findings by Grethen and cols. (30). Type 2 diabetes mellitus was frequent in our study participants, and the degree of obesity varied with the presence of diabetes and use of antidiabetic drugs, as shown in Supplementary Table 2. However, only the use of antihypertensive and hypoglycemic agents has been associated with the occurrence of fractures (probably due to falls) (31). Notably, the exclusion of patients with diabetes in our study did not change the results considerably.

The use of PPIs was associated with an increased prevalence and number of fractures in our population. PPI use was associated with decreased BMD (32) and increased occurrence of fractures, possibly due to the effect of these agents in reducing calcium and magnesium absorption and increasing bone resorption (33). PPIs are commonly prescribed for patients with gastrointestinal adverse effects to antiresorptive agents. However, coadministration of PPIs with oral bisphosphonates may interfere with the effect of the latter, and the risk of osteoporosis treatment failure should be considered when medications from both these groups are combined (34).

Traditional risk factors for fractures, including age and low BMD (19), were significantly associated with the risk of fractures in patients with obesity in the present study. Older individuals who are obese have a greater risk of losing lean mass and BMD when losing weight (21). Also, aging was associated with decreased BMI, which may be due to a decrease in lean mass (35), bone loss, and increased risk of fracture in these cases. Additionally, a large number of comorbidities and polypharmacy in these patients contribute to decreasing both BMI and BMD (22).

The limitations of our study were the inclusion of only a select population among all patients who underwent BMD evaluation in the evaluated period. Also, as a retrospective study, we were unable to collect information regarding lifestyle and physical activity habits or obtain measurements of abdominal waist and body composition. All patients were following up at a tertiary hospital and were, thus, more likely to have a greater number of comorbidities. Also, the equipment weight limit of 120 kg prevented the evaluation of all patients with class 3 obesity. Important strengths of our study include the finding that common risk factors for fractures (BMD, lower BMI value within the obese range) also apply to patients with obesity. Our findings also allow for comparisons with data from other studies, providing information on the influence of BMI on BMD and fracture rate for individuals with obesity in the Brazilian population.

In conclusion, the indications for requesting DXA scanning in patients with obesity in the present study were aligned with the indications for the general population, as recommended by the ISCD. We found in our sample a high prevalence of low bone mass, previous fractures, and risk factors commonly associated with fractures such as age, low BMD, and use of PPIs. Increased BMI positively influenced the BMD of our study participants and was associated with a less frequent history of fractures. The results of this study point out a need to obtain bone densitometry evaluation in patients with risk factors for osteoporosis or fractures, regardless of BMI.

## Appendix

**Supplementary Table 1:** Frequency of comorbidities and use of medications

		Total – n (%)	Men – n (%)	Women – n (%)
Comorbidities				
	Hypertension	490 (79.1%)	39 (8.0%)	451 (92.0%)
	Dyslipidemia	437 (70.5%)	44 (10.1%)	393 (89.9%)
	Diabetes	331 (53.4%)	30 (9.1%)	301 (90.9%)
	Osteopenia/osteoporosis	317 (51.2%)	17 (5.4%)	300 (94.6%)
	Thyroid disease	308 (49.7%)	17 (5.5%)	291 (94.5%)
	Hypogonadism	70 (11.3%)	15 (21.4%)	55 (78.6%)
	Bariatric surgery	68 (10.9%)	5 (7.3%)	63 (92.7%)
	Hyperparathyroidism	60 (9.6%)	5 (8.3%)	55 (91.7%)
	Early menopause	42 (6.7%)	–	42 (6.7%)
Medications				
	Vitamin D	455 (73.5%)	32 (7.0%)	423 (93.0%)
	Antihypertensive drugs	447 (72.2%)	38 (8.5%)	409 (91.5%)
	Statins	399 (64.4%)	36 (9.0%)	363 (91.0%)
	Diuretics	332 (53.6%)	25 (7.5%)	307 (92.5%)
	Antidiabetics	302 (48.7%)	25 (8.3%)	277 (91.7%)
	Calcium supplements	289 (46.6%)	19 (6.6%)	270 (93.4%)
	Antiresorptive agents	163 (23.3%)	7 (4.3%)	156 (95.7%)

N: number.

**Supplementary Table 2:** Rates of different variables according to obesity classification

Variable			Class 1 N (%)	Class 2 N (%)	Class 3 N (%)	p
***Demographic data***						
Age (mean ± SD)			63.77 (±12.25)	61.68 (±11.68)	59.86 (±12.78)	**0.030**
Sex						0.060
	Women		339 (54.76%)	147 (23.75%)	69 (11.14%)	
	Men		43 (6.94%)	19 (3.06%)	2 (0.32%)	
Race						**0.029**
	White		367 (59.20%)	156 (25.20%)	63 (10.17%)	
	Black		15 (2.42%)	10 (1.61%)	7 (1.13%)	
	Asian		0	0	1 (0.16%)	
Weight (mean ± SD)			77.81 (± 8.81)	90.25 (± 10.41)	102.24 (±12.84)	**<0.001**
Height (mean ± SD)			1.55 (±0.07)	1.56 (±0.08)	1.53 (±0.08)	0.240
BMI (mean ± SD)			32 (±1.41)	37 (±3.18)	43 (±2.58)	**<0.001**
Lifestyle habits						
	Current smoking		26 (6.8%)	9 (5.4%)	3 (4.2%)	0.740
	Previous smoking		80 (20.9%)	32 (19.3%)	17 (23.9%)	0.710
	Package-years		6.46 (±14.15)	7.27 (±22.8)	5.14 (±10.54)	0.780
	Alcoholism		7 (1.8%)	2 (1.2%)	2 (2.8%)	0.600
***BMD***						
	Low bone density		279 (73%)	105 (63.2%)	43 (60%)	**0.010**
	Prior low bone density		205 (53.6%)	81 (48.8%)	31 (43.6%)	0.230
		Indication for DXA scanning	326 (±85.3)	137 (±82.5)	55 (±77.4)	0.220
***Medications***						
	Calcium and vitamin D		289 (75%)	130 (78.3%)	50 (70.4%)	0.420
	Vitamin D		281 (73.5%)	125 (75.3%)	49 (69.0%)	0.600
	Antihypertensive drugs		267 (69.8%)	127 (76.5%)	53 (74.6%)	0.250
	Statins		244 (63.8%)	102 (61.4%)	53 (74.6%)	0.140
	Calcium		191 (50%)	74 (44.5%)	24 (33.8%)	**0.034**
	Diuretics		190 (49.7%)	96 (57.8%)	46 (64.8%)	**0.020**
	Antidiabetics		166 (43.4%)	92 (55.4%)	44 (61.9%)	**0.002**
	PPI		137 (35.8%)	63 (37.9%)	26 (36.6%)	0.890
	Levothyroxine		160 (41.8%)	61 (36.7%)	36 (50.7%)	0.130
	Antiresorptive		118 (30.8%)	35 (21.0%)	10 (14.0%)	**0.002**
	Aspirin		96 (25.1%)	57 (34.3%)	28 (39.4%)	**0.010**
	Antidepressant agents		84 (21.9%)	52 (31.3%)	26 (36.6%)	**0.007**
	Insulin		81 (21.2%)	39 (23.5%)	18 (25.3%)	0.670
	Analgesics		56 (14.6%)	25 (15.0%)	11 (15.5%)	0.980
	Corticosteroids		37 (9.6%)	22 (12.2%)	10 (14.0%)	0.330
	Opioids		22 (5.7%)	11 (6.6%)	7 (9.8%)	0.430
	Benzodiazepines		15 (3.9%)	5 (0.3%)	5 (7.0%)	0.320
***Comorbidities***						
	Hypertension		295 (77.2%)	134 (80.7%)	61 (85.9%)	0.210
	Dyslipidemia		275 (71.9%)	113 (68.0%)	49 (69.0%)	0.620
	Menopause		301 (78%)	128 (77%)	57 (80%)	**0.007**
	Diabetes		186 (48.7%)	100 (60.2%)	45 (63.3%)	**0.009**
	Thyroid diseases		191 (50%)	72 (43.3%)	45 (63.3%)	**0.010**
	Asthma		12 (3.1%)	11 (6.6%)	7 (9.8%)	**0.020**
	Osteoporosis		121 (31.6%)	38 (22.9%)	13 (18.3%)	**0.010**
		Other rheumatic disease	116 (30.3%)	49 (29.5%)	22 (30.9%)	0.960
		Cardiovascular disease	79 (20.6%)	32 (19.2%)	20 (28.1%)	0.290
	Fracture		62 (16%)	20 (12%)	5 (7%)	0.090
		Neoplasms	50 (13%)	19 (11.4%)	6 (8.4%)	0.520
		Hypogonadism	44 (11.5%)	17 (10.2%)	9 (12.6%)	0.840
		Early menopause	42 (10.9%)	18 (10.8%)	2 (2.8%)	0.200
	Psychiatric diseases		38 (9.9%)	22 (13.2%)	15 (21.1%)	**0.020**
		Hyperparathyroidism	38 (9.9%)	14 (8.4%)	8 (11.2%)	0.760
	Bariatric surgery		32 (8.3%)	22 (13.2%)	14 (19.7%)	**0.010**
		Kidney disease	30 (7.8%)	7 (4.2%)	7 (9.8%)	0.190
	Thyroidectomy		33 (8.6%)	15 (9.0%)	7 (9.8%)	0.910
		Other endocrine diseases	24 (6.2%)	10 (6.0%)	4 (5.6%)	1.00
		Liver disease	25 (6.5%)	18 (10.8%)	7 (9.8%)	0.190
		Gastrointestinal disease	22 (5.7%)	12 (7.2%)	4 (5.6%)	0.770
		Rheumatoid arthritis	15 (3.9%)	9 (5.4%)	3 (4.2%)	0.700
	Parathyroidectomy		12 (3.1%)	1 (0.6%)	2 (2.8%)	0.190
	COPD		10 (2.6%)	6 (3.6%)	4 (5.6%)	0.330
		Myopathy	4 (1.0%)	3 (1.8%)	1 (1.4%)	0.570
***Laboratory tests***						
		Vitamin D (ng/mL) (mean ± SD)	31.28 (±10.85)	28.47 (±10.11)	28.91 (±9.66)	0.330
		Calcium (mg/gL) (mean ± SD)	9.53 (±0.87)	10.23 (±7.9)	9.58 (±0.54)	0.360
		PTH (pg/dL) (mean ± SD)	81.44 (±65.53)	95.94 (±118.16)	90.05 (±55.16)	0.500
		FT4 (ng/dL) (mean ± SD)	1.1 (±0.24)	1.13 (±0.24)	1.37 (±1.64)	0.590
		TSH (pg/mL) (mean ± SD)	2.76 (±4.22)	2.09 (±1.60)	2.45 (±2.22)	0.700
		HbA1C (%) (mean ± SD)	6.63 (±1.63)	6.56 (±1.67)	6.84 (±1.8)	0.520
		Fasting blood glucose (mg/dL) (mean ± SD)	116 (±40.56)	117 (±39.23)	127 (±49.3)	0.350
		Creatinine (mg/dL) (mean ± SD)	0.94 (±0.38)	0.96 (±0.68)	0.9 (±0.22)	0.720
		Total cholesterol (mg/dL) (mean ± SD)	182.55 (±37.22)	174.32 (±38.06)	174.83 (±34.89)	**0.030**
		LDL cholesterol (mg/dL) (mean ± SD)	107.9 (±38.37)	101 (±33.18)	98.6 (±31.18)	**0.040**
		HDL cholesterol (mg/dL) (mean ± SD)	48.2 (±22.65)	47.42 (±16.02)	47.24 (±11.42)	0.850
		Triglycerides (mg/dL) (mean ± SD)	148.4 (±77.32)	136.1 (±65.23)	138.5 (±56. 32)	0.230
		AST (U/L) (mean ± SD)	21.77 (±16.72)	22.82 (±12.97)	21 (±10.67)	0.480
		ALT (U/L) (mean ± SD)	21.79 (±13)	21.02 (±8.35)	21.12 (±10.67)	0.870
History of fractures			62 (71.3%)	20 (23%)	5 (5.7%)	0.091

Distribution of patients according to obesity class: class 1 (n=382), class 2 (n=166), class 3 (n=71). N: number; SD: standard deviation; BMI: body mass index; BMD: bone mineral density; DXA: dual-energy X-ray absorptiometry; PPI: proton pump inhibitor; COPD: chronic obstructive pulmonary disease; PTH: parathyroid hormone; FT4: free thyroxine; TSH: thyroid-stimulating hormone; HbA1C: hemoglobin A1C; LDL: low-density lipoprotein; HDL: high-density lipoprotein; AST: aspartate aminotransferase; ALT: alanine aminotransferase.
